# Primary Visual Cortex Activity along the Apparent-Motion Trace Reflects Illusory Perception

**DOI:** 10.1371/journal.pbio.0030265

**Published:** 2005-07-19

**Authors:** Lars Muckli, Axel Kohler, Nikolaus Kriegeskorte, Wolf Singer

**Affiliations:** **1**Department of Neurophysiology, Max Planck Institute for Brain Research, Frankfurt am Main, Germany,; **2**Brain Imaging Center Frankfurt, Frankfurt am Main, Germany,; McGill UniversityCanada

## Abstract

The illusion of apparent motion can be induced when visual stimuli are successively presented at different locations. It has been shown in previous studies that motion-sensitive regions in extrastriate cortex are relevant for the processing of apparent motion, but it is unclear whether primary visual cortex (V1) is also involved in the representation of the illusory motion path. We investigated, in human subjects, apparent-motion-related activity in patches of V1 representing locations along the path of illusory stimulus motion using functional magnetic resonance imaging. Here we show that apparent motion caused a blood-oxygenation-level-dependent response along the V1 representations of the apparent-motion path, including regions that were not directly activated by the apparent-motion-inducing stimuli. This response was unaltered when participants had to perform an attention-demanding task that diverted their attention away from the stimulus. With a bistable motion quartet, we confirmed that the activity was related to the conscious perception of movement. Our data suggest that V1 is part of the network that represents the illusory path of apparent motion. The activation in V1 can be explained either by lateral interactions within V1 or by feedback mechanisms from higher visual areas, especially the motion-sensitive human MT/V5 complex.

## Abstract 2

Using fMRI in humans, the authors reveal a clear role for V1 cortex in forming an illusory perception of motion when stationary stimuli are successively flashed in different locations.

## Introduction

Apparent motion can be perceived when two spatially segregated visual stimuli are presented in succession [1]. The illusion persists even when the stimuli are widely separated, a phenomenon called “long-range apparent motion” (here we use the term “apparent motion” to refer to long-range apparent motion) [2]. In order to respond to apparent motion, neurons have to integrate information over a large part of visual space, spanning at least the distance between the two inducing stimuli. Neurons in the middle temporal (MT) area of the macaque have pronounced directional selectivity and receptive-field sizes of up to a 25° visual angle [3–6], which makes them ideally suited for the integration of apparent-motion-inducing stimuli. Several studies have shown that the middle temporal area in the macaque and other primates and its human homolog, the human MT/V5 complex (hMT/V5+), respond to stimulus conditions that induce apparent motion [7–9].

In contrast, receptive-field sizes in early visual areas and, in particular, the primary visual cortex (visual cortex area 1 [V1]) are too small to account for long-range interactions between stimuli [10–13]. The fact that one actually observes spatially resolved movement between the inducing stimuli in apparent motion suggests that there could be a filling-in process in early visual areas that is driven by feedback from extrastriate regions with larger receptive-field sizes. In particular, back-projections from hMT/V5+ have been shown to be relevant for perception of motion and apparent motion [14–17]. A psychophysical study of visual interference along the illusory path of apparent motion has indeed suggested feedback processes acting at the level of V1 as a possible mechanism [18]. Liu et al. [19] have used functional magnetic resonance imaging (fMRI) to test whether the perceptual “filling-in” has an early or late cortical locus. Using a visual display comprising two concentric rings, Liu and colleagues found a late cortical locus (hMT/V5+ and lateral occipital complex) but no activity in the path of apparent motion in early visual areas [19].

We used a different visual display to test the same hypotheses, and we report here about V1 activity along the illusory motion path. In order to investigate the topographic pattern of apparent-motion-related activity in V1, we used fMRI in humans to map the retinotopic representation of apparent motion. We specifically looked for activity in subregions that were not directly activated by the apparent-motion-inducing stimuli. Our results show that there is activity in V1 sites representing the illusory motion path that cannot be explained by the local characteristics of the stimulus alone. The influence of attention was controlled with a demanding center task, in which subjects had to detect numbers in a rapidly changing stream of letters and numbers. Using a bistable motion-quartet stimulus [20,21], we confirmed that the activity in V1 changes as a function of the subjectively perceived motion trace. Considering the separation between the apparent-motion stimuli, we argue that this activity is most likely caused by feedback projections from extrastriate regions with larger and direction-selective receptive fields.

## Results

### Experiment 1

**Figure 1:**
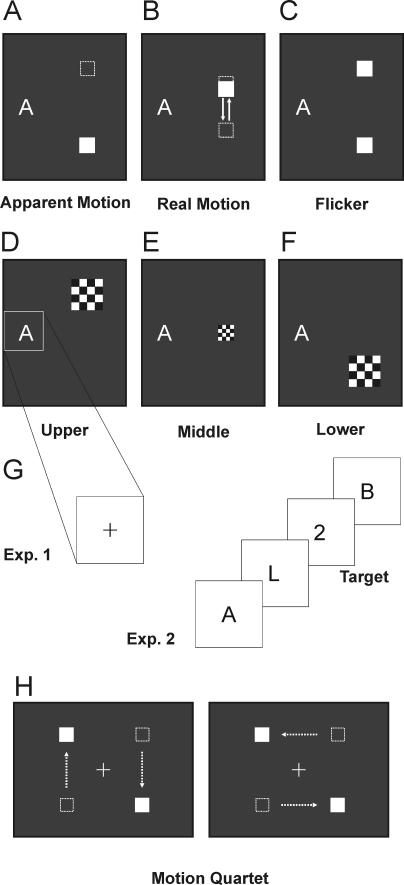
Stimuli Used in Experiments 1 to 3 (A) The apparent-motion stimulus consisted of two white squares (size, 1.8°) blinking in alternation (2.3 Hz). The squares were presented at an eccentricity of 9.5° visual angle (distance between squares, 16.5°). (B) The real-motion stimulus consisted of a white square moving in harmonious oscillation (average speed, 66.5°s^−1^) on the perceived path of apparent motion. The end points of the movement were adjacent to the position of the apparent-motion squares (maximum eccentricity, 8.0°). (C) The flicker control stimulus from experiment 2 was composed of two white squares (1.8°) that were blinking simultaneously at 4.6 Hz. (D–F) For the mapping conditions, high-contrast inverting checkerboards were presented in the upper (D), middle (E), and lower (F) right visual field. Sizes were adjusted according to the cortical magnification factor in V1 to produce activated regions of a similar spatial extent (3.6° for the upper and lower stimuli presented at 9.5° eccentricity, and 1.8° for the middle stimulus presented at 4.7° eccentricity). (G) For the five conditions of experiment 1 (A, B, D–F), subjects saw a white cross at the middle of the screen, which they had to fixate (G, upper part). In experiment 2, a randomly generated stream of letters and digits was presented at 2 Hz in the middle of the screen (G, lower part). Subjects had to fixate the character stream and either passively view the stream or perform a digit-detection task. (H) The motion quartet (experiment 3) was composed of four white squares presented at eccentricities similar to the apparent-motion stimulus. Two squares from diagonally opposite corners were presented at the same time. The motion quartet can be seen in vertical (left part) or horizontal (right part) motion without any changes in the physical characteristics of the stimulus. Subjects had to fixate a white cross in the middle of the screen and had to report the perceived direction of movement (vertical or horizontal).

We determined the extent and borders of early visual areas V1–V3 (Figure S1) in a retinotopic-mapping experiment [8]. In a second session, subjects were presented with five different stimulus conditions (Figure 1). The apparent-motion stimulus (Figure 1A) consisted of two white squares blinking in alternation, presented on a dark screen to the right side of a fixation cross. For mapping of the apparent-motion path, a real-motion stimulus was generated that consisted of a white square moving along the perceived trajectory of the apparent-motion stimulus (Figure 1B). The remaining three conditions consisted of contrast-inverting checkerboards presented at three different locations to map the cortical representation of the apparent-motion-inducing stimuli (upper and lower site, Figure 1D and 1F) and one location on the path of apparent motion (middle, Figure 1E).

To demonstrate specific effects of apparent motion, data were analyzed in experiment 1 using two different approaches. First, we described the spatial distribution of apparent-motion-driven activity in V1 using simple activation maps (apparent motion > baseline) Second, in an explorative manner, we searched for regions of interest (ROIs) within these activity maps that were not activated in any way by the inducing stimuli. It is clearly expected that at the beginning and at the end of the motion streak, locations are also activated by the upper or lower blinking square. Therefore, activity patterns from the apparent-motion map (see above) could be influenced by the effects of the inducing stimuli. The second strategy provides proof of principle that there is activity within the streak that is not activated in any way by the inducing squares.

**Figure 2:**
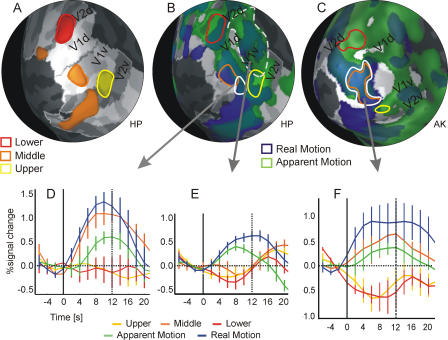
Activation Pattern in V1 for Experiment 1 Medio-posterior view on the inflated left occipital cortex of subject HP (A and B) and subject AK (C). All five subjects are shown in Figure S1. Gray-scale coloring of cortex indicates the extent of retinotopic visual areas (light gray: V1 and V3/VP; dark gray: V2, V4, and V3A) and the gyral pattern (concave surfaces are indicated in darker gray in the respective areas). (A) The cortical representations of the mapping stimuli are marked in color. The maps were obtained by calculating a balanced contrast between the respective mapping condition of interest and the two other mapping conditions (e.g., middle versus upper and lower). The thresholds of *t*-maps were individually adjusted to obtain patches of comparable size in V1: upper (yellow), *t*(600) > 9.3; middle (orange), *t*(600) > 11.9; and lower (red), *t*(600) > 15.1 (all *p* << 0.001). (B) Cortical activation maps for the apparent-motion (green) and real-motion (blue) condition compared to baseline (apparent motion in V1: *t*[600] > 3.6, *p* < 0.0004; real motion in V1: *t*[600] > 18.6, *p* << 0.001). Cortical representations of the mapping stimuli are indicated by colored outlines taken from (A). The solid white line indicates a patch of significant activation (*t*[600] > 3, *p* < 0.003) for the following conjunction of contrasts, which represents the ideal activation pattern: (middle > upper) and (middle > lower) and (apparent motion > upper) and (apparent motion > lower). The dashed white line marks a peripheral region in V1 with significant activation in response to apparent motion and real motion. (C) Same as (B) for subject AK. The white region with the optimal response pattern overlaps largely with the representation of the middle stimulus (in orange; see Figure S1 for details). (D) Event-related BOLD signal change for subject HP plotted over time from the patch outlined by the solid white line in (B) (solid black line indicates stimulus onset, dotted line stimulus offset). (E) Event-related BOLD signal change of the apparent-motion-activated (green) region from the eccentric parts of V1 outlined by the dashed white line in (B) (solid black line indicates stimulus onset, dotted line stimulus offset). This eccentric region responds to real motion and apparent motion but not to the middle stimulus. (F) Same as (D) for subject AK. Error bars correspond to standard errors of the mean.

Following the first strategy (green map in Figures 2 and S1), we found that the activity evoked during apparent motion spanned the region of V1 representing the illusory motion path between the two apparent-motion-inducing stimuli (*t* > 2, *p* < 0.05 for each single-subject analysis). This streak of activity was always located on the real-motion path and, in four out of five subjects, was also activated by the middle mapping stimulus (Figure S1). Moreover, the activation maps for real motion and for apparent motion covered additional parts of V1 peripheral to the direct connection between the end points (Figure 2B and 2E). Following the second strategy, we found in four subjects ROIs within V1 between the mapped end points of the motion path that were activated in the apparent-motion condition but did not respond at all when the upper or lower mapping stimulus was presented alone (Figures 2 and S1).

### Experiment 2

In the second experiment, we wanted to replicate our findings and to control for possible alternative explanations for the apparent-motion-related activity. We added a task that required the subjects to divert their attention to the center of the visual field. We presented a stream of rapidly changing digits and letters instead of the fixation cross and instructed the subjects to respond to the appearance of the digits (see Figure 1G). Separate fMRI scans were acquired while the subjects either performed the attention task or passively viewed the character stream. At the same time, six different conditions were presented in the periphery: five conditions from experiment 1 (real motion, apparent motion, upper, middle, and lower) and an additional control condition, in which two squares were blinking simultaneously at the upper and lower positions (flicker, see Figure 1C).

As in experiment 1, we first described the spatial distribution of apparent-motion-driven activity in V1 using contrast maps (apparent motion > flicker) and searched for ROIs within these activity maps that were not activated in any way by the inducing stimuli. In addition to the two analysis strategies applied in experiment 1, we used a further two approaches that were more objective. As a third strategy, we selected ROIs in individual subjects from the representation of the apparent-motion path by use of the middle mapping condition (conjunction map: middle > upper and middle > lower). In the fourth approach, ROIs were selected by contrasting the real-motion condition with the outer mapping conditions. These ROI-based approaches are comparable to the one followed in the study by Liu et al. [19].

**Figure 3:**
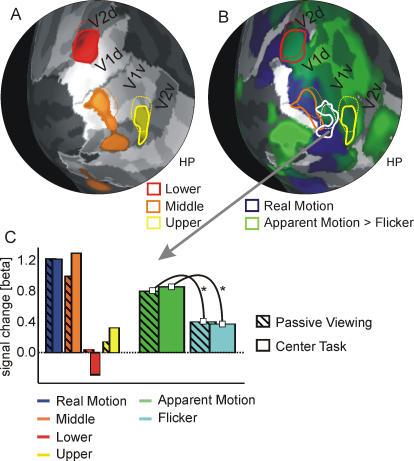
Cortical Activation in V1 for Experiment 2 (A) Left occipital cortex of subject HP (all five subjects are shown in Figure S2) with superimposed contrast maps indicating the cortical representation of the stimulus positions: upper (yellow), *t*(1445) > 15; middle (orange), *t*(1445) > 12; and lower (red), *t*(1445) > 9.3 (all *p* << 0.001). For comparison, the patches from experiment 1 (see Figure 1) are marked with dotted lines. (B) Cortical activation maps for apparent motion (green) and real motion (blue). In this case, apparent motion is contrasted with the flicker control condition (apparent motion > flicker in V1: *t*[1445] > 3.6, *p* < 0.0004); real motion is compared to fixation baseline (real motion > baseline in V1: *t*[1445] > 18.6, *p* << 0.001). The white line indicates an example of a significant (*t* > 2.2, *p* < 0.05) contrast conjunction: (middle > upper) and (middle > lower) and (apparent motion > flicker). (C) BOLD activity profile of the region indicated by the white line in (B). Bars indicate average activity during the respective conditions expressed in beta weights from a GLM analysis.

Using strategies 1 and 2, we replicated the previous findings (*n* = 5, three of whom had already participated in experiment 1). Again, we found apparent-motion-related activity between the end points (green map in Figures 3 and S2) in patches that were overlapping with regions activated by real motion (blue map) and the middle mapping stimulus. Moreover, the activation maps for real motion and for apparent motion covered additional parts of V1 peripheral to the direct connection between the end points. The ROI time courses show that activated regions in the middle of the motion streak responded exclusively to middle mapping, real motion, and apparent motion but not to upper or lower mapping stimulation. The activation of these not directly stimulated areas remained significant in seven out of ten single comparisons when we compared the apparent-motion-related activity to the activity caused by the flicker control condition (Figure S2). In this condition, the end points were stimulated in the same way as during apparent-motion induction, except that the squares were presented simultaneously and did not produce apparent motion (contrast apparent motion versus flicker; *t*[1445] > 2, *p* < 0.05 in the significant single-subject analyses).

**Figure 4:**
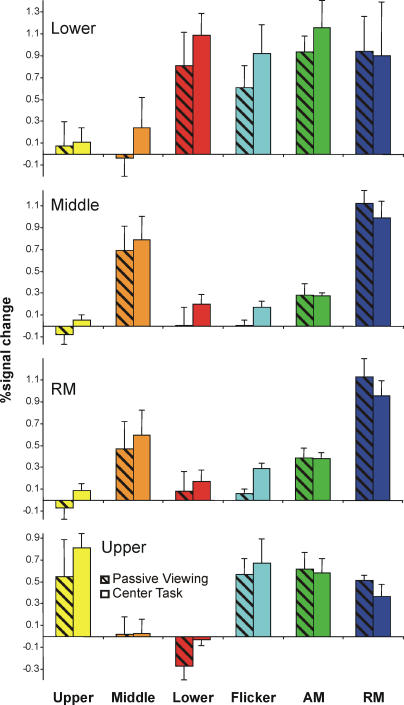
Response Profile for Different ROIs in V1 for Experiment 2 The four panels correspond to four ROIs that were defined in V1 by the respective conditions. To determine the ROIs for the three mapping conditions—upper, middle, and lower—the relevant condition was contrasted with the remaining two mapping conditions in a conjunction analysis (e.g., upper > lower and upper > middle). For the real-motion ROI, the real-motion condition was contrasted with the upper and lower mapping conditions (conjunction analysis). The bars designate percentage signal change of the peak response relative to baseline for the different conditions (see Materials and Methods). The hatched bars are activations from runs with passive viewing only, and the solid bars are from runs with center task. For both the real-motion and middle ROI, the response to the apparent-motion stimulus is significantly larger than the response to the flicker squares. This is not the case for the upper and lower ROIs. Error bars correspond to standard errors of the mean.

Following strategies 3 and 4, we computed event-related averages of V1 activity for different ROIs (Figure 4). The four ROIs were defined separately in individual subjects as parts of V1 that showed a significant response to one of the three mapping conditions (upper, lower, and middle) or to the real-motion condition. The peak response relative to baseline was then compared for the different conditions and *t*-tests were computed across subjects. The ROIs for the upper and lower condition showed the expected response profile. Activation was strong for the respective mapping stimuli as well as for flicker and apparent motion, which were presented at the same location as the mapping conditions. The regions also showed a substantial response to real motion, probably because of larger receptive-field sizes in the periphery and because the real-motion square overlapped with the outer mapping stimuli. There was no significant difference in the responses to the apparent-motion and flicker conditions (*p* > 0.10), suggesting that the two conditions were equivalent in terms of the local activity produced by the inducing stimuli. For the two ROIs on the path of apparent motion, middle mapping, and real motion, the response profile looked different: For both regions, real motion produced the strongest activation, followed by the middle condition. In addition, there was a reliable response to apparent motion that was higher than the activity induced by the flicker squares. This effect decreased when the subjects' attention was focused on the letters–digits discrimination task (Figures 4 and S2) but was still significant (*p* < 0.05). Notably, the decrease was not due to a reduction of apparent-motion-related activity but to an increased activity during the flicker control condition.

**Figure 5:**
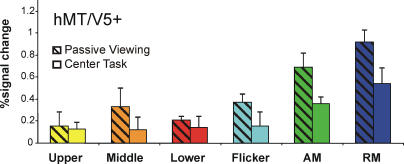
Attentional Modulation of Activity in hMT/V5+ in Experiment 2 Responses to the different conditions in experiment 2 for the region hMT/V5+ are shown. The bars designate percentage signal change of the peak response relative to baseline (see Materials and Methods). The hatched bars are activations from runs with passive viewing only, and the solid bars are from runs with center task. The static conditions—upper, middle, lower, and flicker—induced only low activation in hMT/V5+. In contrast, the response to apparent motion and real motion was higher, corresponding with the motion sensitivity of hMT/V5+. The attentional modulation of the responses was very strong. Under all conditions, the activity was smaller during runs with center task than during runs with passive viewing. Error bars correspond to standard errors of the mean.

It has been demonstrated in previous fMRI studies that spatial attention strongly modulates activation in visual cortical areas down to V1 [22–27]. To validate the effectiveness of our attention-demanding center task, we compared the activation in hMT/V5+ for the different conditions in runs with center task to the activation found with passive viewing (Figure 5). In addition, we looked at the attentional modulation in V1 at the upper and lower locations (see Figure 4). In hMT/V5+, the general activation level was highest for real motion and apparent motion followed by flicker and the mapping stimuli (see Figure 5). For both task conditions, passive-viewing and center task, hMT/V5+ was activated significantly more strongly for apparent motion than for flicker (*t*[7247] > 2.7, *p* < 0.01), in correspondence with the apparent-motion sensitivity of hMT/V5+ [8,9].

We computed an attentional-modulation index (AMI) [26] for the three main conditions of interest: real motion, apparent motion, and flicker. The AMI equals the difference between the responses of the passive-viewing and center task runs normalized by the response of the passive-viewing runs ([passive viewing − center task]/passive viewing). A positive AMI value indicates a reduction in activation for the center task runs. In hMT/V5+, the AMI was 0.57 for flicker, 0.48 for apparent motion, and 0.41 for real motion. The very high AMI values show that the subjects' attention was efficiently diverted from the peripherally presented stimuli. In contrast to the results in hMT/V5+, the pattern of attentional modulation was very different in the V1 ROIs (see Figure 4). Only real motion showed a positive AMI, with 0.20 for the lower ROI and 0.34 for the upper ROI. The other relevant conditions showed an enhanced response with the center task; their AMI values ranged from −0.55 to −0.08.

### Experiment 3

In a third experiment, we presented a bistable apparent-motion stimulus that consisted of four blinking squares (motion quartet; see Figure 1H). The motion quartet induces spontaneous switches between the perceptions of vertical or horizontal apparent motion without any changes in the physical characteristics of the stimulus [20,21]. This allows us to identify activity that is closely related to the conscious perception of apparent motion [9]. We presented a motion quartet with the two squares in the right hemi-field at approximately the same locations as the apparent-motion stimuli in experiments 1 and 2. Subjects (*n* = 6; five subjects from experiment 2) continuously reported their current percept using the left and right response buttons for vertical and horizontal movement, respectively.

**Figure 6:**
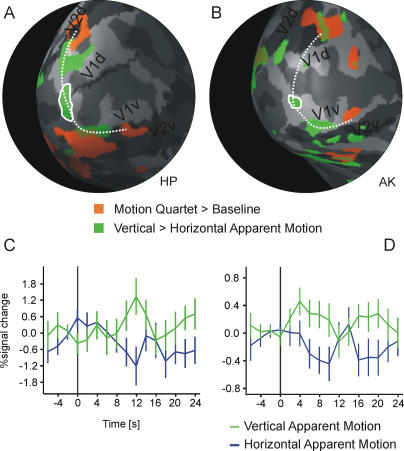
Results from the Bistable Motion Quartet (Experiment 3) (A and B) Medio-posterior view on the inflated left occipital cortex of subject HP (A) and AK (B) (all six subjects are shown in Figure S3). Gray shading indicates the extension of V1 (light gray for V1) and the cortex curvature (dark gray, concave; light gray, convex). Activation maps show the cortical representation of the stimulated locations in red (motion quartet > baseline). Contrast maps in green indicate regions that are more active for vertical apparent motion than for horizontal apparent motion (ROI-based GLM at indicated locations; HP: *t*[301] > 2.5, *p* < 0.02; AK: *t*[708] > 2, *p* < 0.05). The dotted line is a spline-interpolated curve connecting the stimulated locations and the region that is more active during the perception of vertical apparent motion. (This line does not necessarily indicate the path of apparent motion from experiments 1 and 2 since stimulus parameters had to be adjusted.) (C and D) The solid white lines mark regions from which event-related averages were calculated. Event-related averages are shown for subject HP (C) and for subject AK (D). The time courses are aligned to the time point at which the subject indicates a switch in perception (*t* = 0, black line) and are shown for the time period from 4 s before to 24 s after the perceptual switch. The first perceptual period following each stimulation onset is omitted from the analysis (see Materials and Methods). Error bars correspond to standard errors of the mean. For HP, the vertical percept lasted on average for 11.9 s (standard deviation, 10.3 s), the horizontal for 13.4 s (standard deviation, 9.2 s). For AK, the vertical lasted for 14.6 s (standard deviation, 7.1 s), and the horizontal for 16.6 s (standard deviation, 6.7 s).

For experiment 3, we calculated contrast maps that indicate higher activation in response to vertical as compared to horizontal apparent-motion perception. We found patches of activity that showed a selective increase of activity following perceptual switches from horizontal apparent motion to vertical apparent motion in all six subjects (green regions in Figures 6 and S3). These patches were located within V1 between the cortical representations of the inducing stimuli (red regions in Figures 6 and S3) consistent with the cortical representation of the vertical motion streak.

## Discussion

We have shown in three experiments that there is V1 activity that correlates with the perception of long-range apparent motion. We observed activity patterns that corresponded to the expected motion streak. In ROI analyses, the apparent-motion activity was found to be significantly higher than in the flicker control condition and was also unmodulated by attentional set. In an event-related design with the bistable motion quartet, the activity could be closely linked to the conscious perception of apparent motion in response to perceptual switches.

### Horizontal Connections

Our results indicate that there are subregions in V1 that are active during illusory apparent-motion perception even though the inducing stimuli are presented outside the receptive fields of the neurons in these regions. This spreading extra-receptive-field response could be mediated by horizontal connections in V1 [13,28]. However, three arguments render it unlikely that horizontal interactions within V1 are a major source of activity along the illusory motion path. First, the flicker stimuli did not induce a comparable extra-receptive-field response although they should have caused the same local activation as the apparent-motion-inducing stimuli. Second, the horizontal connections in V1 span only short distances that are well below the distance between the representations of the apparent-motion-inducing stimuli [13]. Third, in a motion quartet there is perceptual competition between vertical and horizontal apparent motion, leading to mutually exclusive percepts. There is evidence that this competition is resolved in hMT/V5+ [29].

### Attention and Feedback

A more likely candidate for the apparent-motion-related activity is top-down influence from higher areas [30]. Attention increases the blood-oxygenation-level-dependent (BOLD) signal in early visual areas including V1 [23–25,27,31,32], even in the absence of visual stimulation [26]. However, data from our control experiment show that the apparent-motion-related activation persisted when we diverted the subjects' attention away from the stimuli. Thus, apparent-motion-related activation is not solely due to spatial attention. This suggests an attention-independent but motion-specific filling-in process associated with the illusory motion percept.

The best candidate area for a possible top-down influence on V1 related to processing of apparent motion is hMT/V5+. Several studies have emphasized the important influence that feedback from higher areas has on functions of V1 [30,33–36], and feedback from hMT/V5+ to V1 has been assigned a role in the perception of real motion and apparent motion [14–18]. Neurons in hMT/V5+ have receptive fields large enough to span the distance between the apparent-motion-inducing stimuli, and they respond to apparent motion and real motion in similar ways [8,9,37]. The back-projections from higher to lower cortical areas fan out and can span at least the size of their receptive fields [13,38,39].

We observed illusion-related activity and real-motion-related activity not only on the direct motion path but also in the periphery of the motion streak. Coactivation of more peripheral sections might be a result of feedback activity that is spreading out to larger sections in the periphery. It is particularly those cells that have sufficiently large receptive fields (16.5°) to cover both apparent-motion-inducing stimuli (illusion-related activity and real-motion-related activity) that are expected to be found in more peripheral sections of higher visual areas and are therefore expected to back-project, especially to peripheral parts in early visual areas.

### Previous and Related Findings

Why was the earlier attempt by Liu et al. [19] unsuccessful in finding apparent-motion-related activity in V1? The experimental strategy that they employed was comparable to ours in most aspects, but there was a significant difference in the stimulation material that they used: large rings inducing radial inward–outward apparent motion. The stimulation of large sections of the visual cortex might have induced a complex pattern of excitation and inhibition in directly adjacent parts of V1. Moreover, we showed that in most subjects apparent-motion-related activity is displaced to the periphery. So in the case of inward–outward apparent motion, much of the apparent-motion-related activity might be displaced towards the outer ring.

A number of recent studies have demonstrated a close relationship between activation in V1 and conscious perception of visual stimuli. In these studies, different imaging and electrophysiological methods have been used to investigate the functional properties of V1 during binocular rivalry [40–43], perception of moving phosphenes [15,17], figure–ground segregation [34,44–46], the “line-motion” illusion [28], and color filling-in [47]. In contrast to previous results [48], it was found that V1 activity can be tightly linked to visual awareness. This has been supported in other experiments showing that complex features, such as motion-defined edges [49] and second-order motion [50,51], are represented as early as in V1. The new view on early visual processing has been applied to other sensory systems. Chen et al. [52] demonstrated that correlates of a tactile illusion could already be found in primary somatosensory cortex. In our study, we extended the previous findings to the domain of apparent motion.

Current findings from simultaneous fMRI and electrophysiological recordings in monkeys have suggested that the BOLD signal might be especially sensitive to changes in local field potentials [53]. A more recent study established a tight link between BOLD signal and neuronal synchronization [54]. This could explain why the spiking activity of neurons located along the illusory motion trace has been found unchanged in earlier electrophysiological studies of V1 in macaques [4]. Logothetis and colleagues showed that the BOLD signal is correlated with an increase in neuronal activity but further suggested that it primarily reflects the input of a cortical area and its local intra-cortical processing, including excitatory and inhibitory inter-neurons [55]. Our data are consistent with the interpretation that at the level of hMT/V5+, motion features are extracted from neurons with sufficiently large receptive fields to cover both apparent-motion-inducing stimuli. Feedback from hMT/V5+ could cause synaptic processes at the level of V1 that produce a BOLD response without causing major increases in spiking activity. Whether and how interactions between hMT/V5+ and V1 contribute to the perception of apparent motion remains to be investigated in future studies.

## Materials and Methods

### Subjects

Eight subjects (six male) participated in the fMRI experiments; their mean age was 29.2 y (range, 22–34 y). All subjects had normal or corrected-to-normal (one subject) vision. The participants received information on fMRI and a questionnaire to check for potential health risks and contraindications. Volunteers gave their informed consent after being introduced to the procedure in accordance with the Helsinki declaration (www.wma.net/e/ethicsunit/helsinki.htm). All subjects participated in retinotopic mapping. Five subjects participated in experiment 1, five subjects in experiment 2, and six subjects in experiment 3. Of the subjects who participated in experiment 1, three also participated in experiments 2 and 3. Of the subjects who participated in experiment 2, five also participated in experiment 3.

### Stimuli

Stimuli were generated with custom-made software based on the Microsoft DirectX library (StimulDX, Brain Innovation, Maastricht, The Netherlands). The stimuli were back-projected onto a frosted screen with a liquid-crystal-display projector (VPL PX 20, Sony, Tokyo, Japan) and a custom-made lens. Subjects viewed the screen through a mirror. Mirror and projection screen were fixed onto the head coil. Seven different types of stimuli were used in the experiments (see Figure 1; stimulus in Figure 1C was used only in experiment 2 and stimulus in Figure 1H only in experiment 3). The apparent-motion stimulus (see Figure 1A) consisted of two white blinking squares (size, 1.8° visual angle) presented in alternation on a dark screen to the right side of the fixation cross. The squares were presented at an eccentricity of 9.5° visual angle (distance between squares, 16.5°). Stimulus duration was 150 ms with an inter-stimulus interval of 67 ms, corresponding to a presentation frequency of 2.3 Hz. This specific frequency was chosen based on previous results showing that the perception of apparent motion is strongest in an envelope from 2 to 3 Hz [56,57]. A real-motion stimulus (see Figure 1B) was generated by presenting a white square in harmonious oscillation (location on the path equals the sine of time) on the perceived path of the apparent-motion stimulus (size, 1.8° visual angle).

The end points of the oscillation were directly adjacent to the position of the apparent-motion-inducing squares. The maximum eccentricity of the squares was 8.0° and the distance between the end points of the motion path 13.3° (average speed, 66.5°s^−1^). Two static stimuli were used to map the locations where the apparent-motion squares were presented (upper and lower checkerboard in Figure 1D and 1F) and one static stimulus to map the center region of the apparent-motion path (middle checkerboard in Figure 1E). The middle stimulus was located halfway between the two squares of the apparent-motion stimulus at an eccentricity of 5.0° (size, 1.8°). The static stimuli were checkerboards consisting of a 4 × 4 matrix of alternating black and white squares. The checkerboards inverted their contrast every 100 ms, corresponding to a presentation frequency of 5 Hz. The size of the squares was 3.6° (upper and lower) and 1.8° (middle).

In experiment 2, we used an additional control stimulus (see Figure 1C) that was identical to the apparent-motion stimulus except that the two squares were presented in synchrony, resulting in a presentation frequency of 4.6 Hz. To control for attention effects, we also introduced a demanding center task [58]. Subjects saw a stream of alphanumeric characters instead of the fixation cross and had to press a button whenever they detected a numeric character (see Figure 1G). The presentation frequency of the characters was 2 Hz and targets appeared with a probability of 0.125.

In experiment 3, we presented four blinking squares in a rectangular configuration in which the diagonally opposing dots blinked simultaneously (see Figure 1H). We presented two dots at approximately the same positions in the right visual field as in experiments 1 and 2. Two further dots were presented in the left visual field and were therefore not processed by ipsilateral V1 [59]. We adjusted for each subject the vertical distance of the squares in order to make the stimulus bistable.

### Procedure

Each of the conditions in experiment 1 (five conditions) and experiment 2 (six conditions) was presented for approximately 12.5 s (six volumes) in a block design, separated from the next block by a fixation period of the same length. A complete presentation cycle consisted of the following sequence of conditions (with intervening fixations): middle, apparent motion, upper, real motion, and lower (60 volumes) in experiment 1; and middle, apparent motion, upper, real motion, lower, and flicker (72 volumes) in experiment 2. In each following cycle, the presentation order of the conditions was reversed with respect to the preceding cycle, providing a control for serial-order effects. A complete scan comprised five cycles of experimental conditions plus an additional eight volumes of fixation at the beginning of the scan (308 volumes for experiment 1 and 368 volumes for experiment 2). In experiment 1, two complete scans were acquired. Subjects were asked to fixate and be attentive during the scans. In experiment 2, four scans were carried out with each subject. In two scans of experiment 2, the subjects had to perform the attention task, while in the other two scans they only had to fixate the alphanumeric stream of the attention task. In the two runs of experiment 3, we presented the bistable motion quartet five times for 125 s (60 volumes each). The five presentation periods were separated by 21 s (ten volumes) of fixation. During the motion-quartet trials, subjects had to indicate their current percept (vertical versus horizontal motion) by continuously pressing one of two buttons with their right index and middle fingers. They were instructed to apply a strict criterion for changes of perception.

### Imaging

fMRI scanning was performed on a 1.5 Tesla Siemens Magnetom Vision scanner (Siemens, Erlangen, Germany) at the University Clinic in Frankfurt am Main. A gradient-recalled echo-planar-imaging sequence was used with the following parameters: 16 or 18 slices, oriented approximately in parallel to the anterior commissure–posterior commissure plane; recording time for one volume (TR), 2,081 ms; TE, 60 ms; FA, 90°; FOV, 210 mm; in-plane resolution, 3.44 × 3.44 mm; slice thickness, 4 mm; and gap thickness, 0.4 mm. In addition, a T1-weighted anatomical scan was acquired for all subjects using a Siemens fast low-angle-shot (FLASH) sequence (isotropic voxel size, 1 mm^3^).

### Data analysis

Data were analyzed using the BrainVoyager 4.9 software package (Brain Innovation). The first four volumes of each scan were discarded to preclude T1 saturation effects. Preprocessing of the functional data included the following steps: (i) three-dimensional motion correction using the Levenberg-Marquardt algorithm, (ii) linear-trend removal and temporal high-pass filtering at 0.01 Hz, and (iii) slice-scan-time correction with sinc interpolation.

The statistical analysis was performed with multiple linear regression. For every voxel, the time course was regressed on a set of dummy-coded predictors representing the experimental conditions. To account for the shape and delay of the hemodynamic response [60], the predictor time courses (box-car functions) were convolved with a gamma-variate function.

With the data from experiment 2, five ROIs were defined in individual subjects for a more detailed analysis of activation patterns in V1 and hMT/V5+. The three mapping conditions—upper, middle, and lower—and the real-motion condition were used to determine four ROIs in V1. We used conjunction analyses to effectively restrict the ROIs to parts of V1 with a specific functional profile. For example, to find regions responding specifically to the lower mapping, the activation produced by the lower stimulus had to be significantly higher than the response to the middle as well as the upper stimulus. For the real-motion ROI in V1, the real-motion condition was contrasted with the upper and lower condition. The threshold was set to a Bonferroni-corrected *p*-value of 0.1. The hMT/V5+ ROI was defined by a balanced contrast between real motion and the three mapping conditions (upper, middle, and lower). For four subjects, *p*-values were set to less than 0.2 (Bonferroni-corrected). In one subject only, a more liberal significance level of *p* < 0.002 (uncorrected) had to be used.

To ensure that the increases in event-related activity were not influenced by the preceding conditions, we inspected the event-related time-course averages (see Figures 2 and S1), which are aligned to an average baseline of 0% signal change for the period of 4 s before stimulation onset. The activation patterns for the ROIs were visualized by plotting the percentage signal change of the peak response (corresponding to the average of the three time points around 8 s, 10 s, and 12 s) for the different conditions. The measurements with and without center task were analyzed separately. Statistical significance was assessed across subjects to validate the reliability of the effects in our group of participants.

For experiment 3, we mapped the cortical representation of our inducing stimuli by a general linear model (GLM) analysis in which we looked for higher activity during motion-quartet stimulation as compared to baseline (mapping of all stimulus locations). In addition, we identified regions with higher activation for perceived vertical motion than for perceived horizontal motion and baseline (conjunction analysis). From these regions, we extracted event-related time courses to visualize the activation changes due to the perceptual switches form horizontal to vertical motion. To avoid unspecific stimulus-onset effects, the first perceptual phase of each stimulation block was excluded from the analysis.

### Retinotopic mapping

Phase-encoded retinotopic mapping was assessed in each subject and included mapping of eccentricity and polar angle [8,61–63]. In the eccentricity-mapping experiment, black and white checkerboard patterns were presented in a ring-shaped configuration and were flickered at a rate of 4 Hz. The ring started with a radius of 1° and slowly expanded to a radius of 12° within 96 s. In the polar-angle mapping experiment, the checkerboard pattern consisted of a ray-shaped disk segment subtending 22.5° of polar angle. The ray started at the right horizontal meridian and slowly rotated clockwise for a full cycle of 360° within 96 s. Each mapping experiment consisted of seven repetitions of a full expansion or ten repetitions of rotation, respectively, with each cycle lasting for 64 s.

The analysis of the retinotopic-mapping experiment was conducted by the use of a cross-correlation analysis. We used the predicted hemodynamic signal time course for the first 1/8 of a stimulation cycle (corresponding to a 45° visual angle in the polar mapping experiment) and shifted this reference function successively in time (time steps correspond to the recording time for one volume) [63]. Sites activated at particular eccentricities and polar angles were identified through selecting the lag value that resulted in the highest cross-correlation value for a particular voxel. The obtained lag values at particular voxels were encoded in pseudo-color on corresponding surface patches (triangles) of the reconstructed cortical sheet. Based on the polar-angle mapping experiment, the boundaries of retinotopic cortical areas V1, V2, V3, VP, V3A, and V4v were estimated manually on the inflated cortical surface and colored in shades of light and dark gray.

### Cortical-surface reconstruction

The recorded high-resolution T1-weighted three-dimensional recordings were used for surface reconstruction of both cortical hemispheres of each subject [64]. The white/gray-matter border was segmented with a region-growing method preceded by inhomogeneity correction of signal intensity across space. The borders of the two resulting segmented subvolumes were tessellated to produce a surface reconstruction of the left hemisphere. The resulting surface was used as the reference mesh for projecting functional data on inflated representations. A morphed surface always possesses a link to the folded reference mesh so that functional data can be shown at the correct location on folded as well as inflated representations. This link was also used to keep geometric distortions to a minimum during inflation and flattening through inclusion of a morphing force that keeps the distances between vertices and the area of each triangle of the morphed surface as close as possible to the respective values of the folded reference mesh.

## Supporting Information

Figure S1 Occipital BOLD Activation of Five Subjects in Experiment 1 Color coding for subjects HP, MN, AK, DL, and SW for (B) to (D) is identical to the coding of Figure 2. In addition, maps from a retinotopic-mapping experiment are provided in (A). Gray-scale coloring of cortex indicates the extent of retinotopic visual areas (light gray: V1 and V3/VP; dark gray: V2, V4, and V3A) and the gyral pattern. Borders between visual regions are marked with black lines (A–C).
(A) Retinotopic mapping of early visual areas. Phase-encoded activation maps show regions responding to stimuli in the lower-right quadrant (green to blue) and upper-right quadrant (red to yellow) of the visual field. Red color indicates the representation of the upper vertical meridian, blue and yellow the horizontal meridian, and green the lower vertical meridian. The overlaid outlines (red, orange, and yellow) are from (B) and indicate the cortical representation of the mapping stimuli used in experiment 1.
(B) The cortical representations of the mapping stimuli are marked in color. The maps were obtained by calculating a balanced contrast between the respective mapping condition of interest and the two other mapping conditions (e.g., middle versus upper and lower). The thresholds of *t*-maps were individually adjusted to obtain patches of comparable size in V1: upper (yellow), *t*(600) > 3.8, *p* < 0.0002 (HP > 9.3; MN > 4.0; AK > 4.1; DL > 5.8; SW > 3.8); middle (orange), *t*(600) > 3.2, *p* < 0.002 (HP > 11.9; MN > 5.8; AK > 3.2; DL > 5.6; SW > 6.2); and lower (red), *t*(600) > 4.3, *p* < 0.00003 (HP > 15.1; MN > 8.1; AK > 4.6; DL > 5.6; SW > 4.3).
(C) Cortical activation maps for the apparent-motion (green) and real-motion (blue) conditions compared to baseline—apparent motion in V1: *t*(600) > 2.2, *p* < 0.03 (HP > 3.6; MN > 4.5; AK > 2.2; DL > 8.6; SW > 5.9); real motion in V1: *t*(600) > 5.5, *p* << 0.001 (HP > 18.6; MN > 9.6; AK > 5.5; DL > 8.0; SW > 8.0). Cortical representations of the mapping stimuli are indicated by colored outlines taken from (B). The solid white line indicates a patch of significant activation (*t*[600] > 2.7, *p* < 0.008 [HP > 3.0; MN > 4.1; AK > 2.9; DL > 2.7; SW > 3.4]) for the following conjunction of contrasts, which represents the ideal activation pattern: (middle > upper) and (middle > lower) and (apparent motion > upper) and (apparent motion > lower).
(D) Event-related BOLD signal change plotted over time (from 6 s before to 24 s after stimulus onset) from the respective patch outlined by the solid white line in panel (C). Error bars correspond to standard errors of the mean.
(2.5 MB ZIP).

Figure S2 Occipital BOLD Activation of Five Subjects in Experiment 2 Color coding for subjects HP, MN, AK, LM, and MW is identical to the coding for HP described in Figure 3.
(A) Left occipital hemispheres with superimposed contrast maps indicating the cortical representation of the stimulus positions: upper (yellow), *t*(1445) > 8.3, *p* << 0.001 (HP > 9.3; MN > 12.4; AK > 8.9; LM > 11.4; MW > 8.3), middle (orange), *t*(1445) > 4, *p* << 0.001 (HP > 11.9; MN > 10.4; AK > 4.0; LM > 11.4; MW > 5.9), and lower (red), *t*(1445) > 4.2, *p* << 0.001 (HP > 15.1; MN > 11.0; AK > 11.0; LM > 6.3; MW > 4.2). For comparison, the patches from experiment 1 are marked with dotted lines.
(B) Cortical activation maps for apparent motion (green) and real motion (blue). In this case, apparent motion is contrasted with the flicker control condition (apparent motion > flicker in V1: *t*[1445] > 2.1, *p* < 0.05 [HP > 3.6; MN > 2.4; AK > 3.4; LM > 3.0; MW > 2.6]); real motion is compared to fixation baseline (real motion > baseline in V1: *t*[1445] > 10.1, *p* << 0.001 [HP > 18.6; MN > 20.3; AK > 10.1; LM > 12.6; MW > 11.1]). The white line in (B) indicates the regions from which examples of BOLD activity were taken and presented in (C). Bars indicate average activity during the respective conditions expressed in beta weights from a GLM analysis. Differences between apparent motion and flicker were analyzed separately for runs in which subjects performed the center task (solid bar) and runs in which subjects viewed passively (hatched bar). Although the overall apparent motion-versus-flicker contrasts were significant in each example (*p* < 0.05), not all of the more specific subcomparisons reached the significance level (*p* < 0.05; significant contrasts are marked with asterisks; all *p*-values are corrected for serial correlation).
(1.4 MB ZIP).

Figure S3 Group Results from Bistable Motion Quartet (Experiment 3) Results of experiment 3 are shown on medio-posterior views of the inflated left occipital cortex of all six subjects ([A and B] LM, HP, and AK; [C and D] VV, MW, and MN). Color coding for subjects LM, VV, MW, and MN is identical to the coding for HP and AK described in Figure 6.
(A and C) Activation maps show the cortical representation of the stimulated locations in red (motion quartet > baseline). Contrast maps in green indicate regions that are more active for vertical apparent motion than for horizontal apparent motion (ROI-based GLM at locations indicated by the white line—LM: *t*[708] > 3.3, *p* < 0.001; HP: *t*[301] > 2.5, *p* < 0.02; AK: *t*[708] > 2.0, *p* < 0.05; VV: *t*[708] > 2.6, *p* < 0.01; MW: *t*[353] > 3.0, *p* < 0.01; MN: *t*[708] > 2.3, *p* < 0.02). The dotted line is a spline-interpolated curve connecting the stimulated locations and the region that is more active during the perception of vertical apparent motion. (This line does not necessarily indicate the path of apparent motion from experiments 1 and 2 since stimulus parameters had to be adjusted.) The solid white lines mark regions from which event-related averages were calculated.
(B and D) Event-related averages are aligned to the time point at which the subjects indicated a switch in perception (*t* = 0, black line). The first perceptual period following each stimulation onset is omitted from the analysis (see Materials and Methods). Error bars correspond to standard errors of the mean.
(856 KB ZIP).

## Abbreviations

AMI: attentional-modulation index
BOLD: blood-oxygenation-level-dependent
fMRI: functional magnetic resonance imaging
GLM: general linear model
hMT/V5+: human MT/V5 complex
ROI: region of interest
V[number]: visual cortex area [number]
